# The Spectrum of Congenital Heart Disease in Children in the Andaman and Nicobar Islands: A Five-Year Retrospective Study

**DOI:** 10.7759/cureus.29109

**Published:** 2022-09-13

**Authors:** Ritu Singh, Narayanan Rajaram Tawker

**Affiliations:** 1 Pediatrics, Andaman and Nicobar Islands Institute of Medical Sciences (ANIIMS), Port Blair, IND; 2 Anesthesia and Critical Care, Andaman and Nicobar Islands Institute of Medical Sciences (ANIIMS), Port Blair, IND

**Keywords:** pediatrics, andaman and nicobar islands, india, epidemiology, congenital heart defects

## Abstract

Introduction: Congenital heart disease (CHD) is an abnormality in the structure or function of the cardio-circulatory system present at birth but more often diagnosed subsequently. CHD is the most common (28%) major congenital anomaly and thus signifies a major global health problem. The primary objective is to estimate the frequency and pattern of CHD in children in the Andaman and Nicobar Islands (India).

Methods: We did a hospital-based retrospective observational study. The hospital case records of all children belonging to the age group of 0 to 12 years with newly diagnosed CHD were reviewed for the five years from January 1, 2016 to December 31, 2020. The clinical, demographic, and echocardiogram details were retrieved, and descriptive analysis was done using the Statistical Package for the Social Sciences (SPSS) for Windows Version 26 (IBM, Chicago, USA).

Results: A total of 201 (12.8 per 1000) children were newly diagnosed with CHD (out of a total of 15592 children). There were 110 (54.7%) boys and 91 (45.3%) girls in the age group of 0 to 144 months (mean ± SD: 13.86±27.13). The ventricular septal defect (VSD) is the most common congenital heart defect, accounting for 25.4% of all CHD cases. The most common cyanotic CHD was tetralogy of Fallot (TOF), comprising 8% of the total cases.

Conclusion: The spectrum of CHD in our study was largely similar to pre-existing literature. Although most of the CHDs were detected during infancy, a higher proportion of complex lesions in our study group resulted in adverse outcomes, even in surgically managed cases.

## Introduction

Congenital heart disease (CHD) is an abnormality in the structure or function of the cardio-circulatory system present at birth, even if diagnosed later [1]. It varies in severity, oc­curring from communications between cavities that spontaneously regress to major malformations that even require several procedures, by catheterization or by surgical means. It may result in mortality in the intrauterine, childhood, or adulthood period [2]. CHD is the most common (28%) major congenital anomaly and thus signifies a major global health problem [3]. The birth prevalence of CHD varies among studies worldwide and is mostly reported between 8 and 12 per 1000 [4,5]. With a prevalence of 9 per 1000, approximately 1.35 million newborns are born with CHD every year globally [5]. The prevalence of CHD in India in recent studies reports an increased prevalence of 8.5-13.6 per 1000 children [6,7]. In India, CHD contributes significantly to infant mortality (10%) [8]. The common morbidities reported in children with CHD include developmental delay and cognitive deficits (20-30%) [9,10]. A significant proportion of children born with CHD may lead a normal, productive life if diagnosed early and appropriate medical/surgical intervention is instituted. Thus, early detection of CHD and timely intervention are important for a better outcome in these children [11,12]. The epidemiology of CHD in children in the Andaman and Nicobar Islands was upto this time not researched. Therefore, we undertook this retrospective, observational, hospital-based study to estimate the frequency and pattern of CHD in children attending the only referral hospital in these Islands. The assessment of the frequency and pattern of CHD will help to formulate effective preventive strategies and improve management.

## Materials and methods

We obtained permission to perform this study from the Institutional Ethics Committee (Andaman & Nicobar Islands Institute of Medical Sciences) via letter no. ANIIMS/IEC/2021-22/57. Informed consent was waived off as the data was collected retrospectively from the hospital medical records. All measures to protect the privacy and confidentiality of the study subjects were taken. In this retrospective hospital-based study, we reviewed the hospital case records of all children with newly diagnosed CHD, admitted to the Neonatal Intensive Care Unit (NICU) and Paediatric Ward of the Andaman & Nicobar Islands Institute of Medical Sciences (ANIIMS) and GB Pant Hospital, during the period January 1, 2016 to December 31, 2020. Those children in the age group 0 to 12 years who were suspected of CHD based on clinical/chest X-ray/electro-cardiographic (ECG) findings and once confirmed by 2D-echocardiogram, were included in the study. The children diagnosed with acquired heart disease (e.g., rheumatic heart disease - RHD) and mitral valve prolapse were excluded. The newborns with patent foramen ovale (PFO) and premature neonates with patent ductus arteriosus (PDA) were excluded if they closed spontaneously during the hospital stay or during follow-up (by managing conservatively). A standard pretested validated proforma used during the initial evaluation of every child who gets admitted to our hospital was used, which contains detailed socio-demographic data, history, and clinical examination findings along with chest X-ray, ECG, and echocardiogram (ECHO) findings (as applicable). These data were used for this retrospective study. The spectrum of various CHDs was then analyzed based on the age of presentation, gender distribution, and clinical presentation. The diagnosis was established using a cardiovascular ultrasound system-the GE Healthcare Vivid S6 with a paediatric high frequency (6-8 Hz) probe. Echocardiography including two-dimensional, color, pulse wave, and continuous wave imaging was performed. The details of the study were recorded in a proforma, and the data were generated in an MS Excel sheet (Microsoft® Corp., Redmond, WA). Descriptive analysis was done using the Statistical Package for the Social Sciences (SPSS) for Windows Version 26 (IBM, Chicago, IL).

## Results

There were a total of 15,592 children admitted to the NICU and paediatrics ward from birth to 12 years of age during the five-year study period. Out of these, 201 (12.8 per 1000) were newly diagnosed with CHD. There were 110 (54.7%) boys and 91 (45.3%) girls in the age group of 0 to 144 months (mean ± SD: 13.86±27.13). In the majority of CHDs, (ventricular septal defect - VSD, atrial septal defect - ASD, coarctation of aorta - CoA, tricuspid atresia - TA, Ebstein's anomaly - EA, double outlet right ventricle - DORV, others), there was a male preponderance. In a few CHDs (atrioventricular septal defect - AVSD, pulmonary stenosis - PS, total anomalous pulmonary venous connection - TAPVC, bicuspid aortic valve - BAV), there was a female preponderance, whereas, with PDA, tetralogy of Fallot - TOF, and persistent truncus arteriosus - PTA, there was no gender disparity. Acyanotic heart defects were 141 (70.1%) of the total heart defects, whereas the contribution of cyanotic heart defects was 60 (29.9%). The age and gender distribution of participants in each of these groups are described in Table 1.

**Table 1:** Age and gender distribution of study participants

Age (months)	Type of congenital heart defect (CHD)		
	Acyanotic	Cyanotic	Total
0–12	105	46	151
13–36	18	8	26
37–96	8	3	11
97–108	7	3	10
109–144	3	0	3
Sex			
Male	77	33	110
Female	64	27	91
Total	141	60	201

Out of the 141 children in the acyanotic group, VSD was the most common, whereas in the cyanotic group, TOF was the most common. The frequency, age at diagnosis, and gender distribution of individual CHDs are described in Table 2.

**Table 2:** Spectrum of CHD as per ages of presentation and sex n: number of children; CHD: congenital heart defect; VSD: ventricular septal defect; ASD: atrial septal defect; PDA: patent ductus arteriosus; AVSD: AV septal defect; CoA: coarctation of aorta; AS: aortic stenosis; BAV: bicuspid aortic valve; TOF: tetralogy of Fallot; TGA: transposition of great arteries; TA: tricuspid atresia; T/PAPVC: total/partial anomalous pulmonary venous connection; PTA: persistent truncus arteriosus; EA: Ebstein’s anomaly; DORV: double outlet right ventricle; PS: pulmonary stenosis.

Type of CHD	n (%)	Sex		Age (in months)				
		M	F	0–12	13–36	37–96	97–108	109–144
Acyanotic	141(70.1)	77	64	105	18	8	7	3
VSD	51(25.4)	32	19	43	7	1	0	0
ASD	38(18.9)	20	18	22	7	3	5	1
PDA	18(9)	9	9	15	1	2	0	0
AVSD	4(2)	1	3	3	1	0	0	0
Cardiomyopathy	4(2)	3	1	3	0	0	0	1
CoA	3(1.5)	2	1	3	0	0	0	0
AS	3(1.5)	3	0	3	0	0	0	0
BAV	1(0.5)	0	1	0	0	1	0	0
Others	19(9.5)	7	12	13	2	1	2	1
Cyanotic	59(29.9)	33	27	46	8	3	3	0
TOF	16(8)	8	8	6	5	2	3	0
TGA	4(2)	1	3	4	0	0	0	0
TA	1(0.5)	1	0	1	0	0	0	0
T/PAPVC	7(3.5)	3	4	7	0	0	0	0
PTA	2(1)	1	1	2	0	0	0	0
EA	1(0.5)	1	0	0	1	0	0	0
DORV	3(1.5)	3	0	3	0	0	0	0
PS	9(4.5)	4	5	7	2	0	0	0
Others	17(8.5)	11	6	16	0	1	0	0
Total	201	110	91	151	26	11	10	3

The combination of different heart lesions (labeled as ‘Others’) was found in 17(8.5%). Most children with acyanotic heart lesions are diagnosed during infancy. The children in the cyanotic group were also diagnosed mostly during infancy. However, TOF was recently diagnosed in older children as well.

Among the acyanotic group, feeding difficulty was the most common presenting complaint in 65(45.7%) children. The next common presentations were breathing difficulty in 56(39.4%), recurrent chest infection in 44(30.9%), cough, and poor weight gain in 36(25.7%) each. In the cyanotic group, breathing difficulty was the most common presentation in 44(48.3%), followed by cyanosis in 34(37.3%). The common physical signs on examination, in the acyanotic group, included murmur in 82(57.7%), tachycardia in 64(45%), and tachypnea in 61(42.9%). In the cyanotic group, common symptoms included cyanosis in 49(83%), tachypnea in 39(66.1%), murmur in 18(30.5%), and clubbing in 10(16.9%) cases.

The most common associated syndrome was Down syndrome, in 3% of the patients. Out of 201 children, 119(59.2%) were managed medically, and the rest surgically. On one year of follow-up, mortality occurred in 36(17.9%) cases, as can be seen in Table 3.

**Table 3:** Management and outcome of children with CHD in various age groups

Age (in months)						Total
Management	0-12	13-36	37-96	97-108	109-144	
Medical (total)	97	13	3	4	2	119
Medical (expired)	26	0	0	0	0	26
Surgical (total)	54	13	8	6	1	82
Surgical (expired)	9	1	0	0	0	10
Outcome						
Survival (total)	116	25	11	10	3	165
Expired (total)	35	1	0	0	0	36

Of the children who expired, 26 were medically managed and 10 were surgically managed.

## Discussion

Congenital heart disease is a common problem in the pediatric age group and leads to significant morbidity and mortality in children. The frequency and pattern of CHD in our study group were compared with the findings of other studies in India and abroad.

There were 201 cases of CHD, of which 110 were male (54.7%) and 91(45.3%) females. Among the total cases, acyanotic heart defects were 141(70.1%), whereas the contribution of cyanotic heart defects was 60(29.8%). Among the acyanotic, 77 were males and 64 were females, whereas in the cyanotic group, 33 were males and 27 were females. Similar findings were reported in another study done in Pakistan [13]. In two related studies done in Africa by Chelo et al. [14] in Cameroon and by Otaigbe and Tabansi [15] in Nigeria, the proportion of acyanotic heart lesions was well above 80%.

In our study, 151(75%) cases of CHD were diagnosed during infancy, 26(12.9%) between 13-36 months, and 21(10.3%) between 97 and 144 months. In a similar study [13] on patients from birth to 10 years of age with clinical and ECHO evidence of CHD, more than two-thirds (71%) of the patients were <1 year of age. Shah et al. [16] in their study showed that 39 cases (46.4%) were presented between 1 and 12 months. Other similar studies from India reported detection rates of 69.7% [17], 40.25% [18], and 46.9% [19] in the age group of one month to one year. The higher rate of detection in our setup could be explained by either a higher proportion of critical lesions or early clinical suspicion by doctors at the periphery.

Approximately 84.3% of children born with VSD were diagnosed within the first year of life, whereas the majority of children suffering from TOF seen in our center (62.5%) were diagnosed beyond the first year of life. Zeuchner et al. [20] reported similar findings in Tanzania. But, they observed that there was a female preponderance (M:F ratio of 1:1.17) in Tanzania. Our study showed male preponderance with an M:F ratio of 1.2:1, which was not significantly different. A similar retrospective study by Kapoor et al. in his retrospective study observed a higher prevalence of most types of CHD in male children from 0 to 15 years of age [21].

Some studies [17,18,22] showed a higher male preponderance of 2.08:1, 2:1, and 1.2:1, respectively. This higher disparity in some studies could be due to high health-seeking behavior in parents of male children. In contrast, Amro [23] and Khan et al. [13] did not find much gender disparity.

The clinical presentation of CHD varies according to the type and severity of the defect. During infancy and early childhood, the usual presenting features are cyanosis, digital clubbing, murmur, syncope, squatting, heart failure, arrhythmia, and failure to thrive.

The most common presenting symptom in our study was breathing difficulty, seen in 100 (49.7%) children, followed by feeding difficulty in 81 (40.2%) cases. Poor weight gain and recurrent chest infections were the next common symptoms. A similar finding of breathlessness as the most common symptom was reported by Sharmin et al. [24]. However, a few other Indian studies reported recurrent respiratory infections [25] and fever [17] as the most common symptoms.

The common examination findings in our study included tachypnea and murmur in 100(49.7%) patients, each followed by tachycardia in 87(43.2%). In another study [26], tachycardia (88%) followed by tachycardia (76%) were reported as the most common signs [26].

The presence of a heart murmur was by far the most frequent sign (84.8%, 81.3%) found in numerous studies [18] in India and abroad [14].

In our study, the most common congenital heart defect was VSD, accounting for 25.4% of CHD cases and correlating well with the reported range of 21-53% in the literature [18,19,21,27-30] ASD was the second most common CHD in our study, comprising 18.9%. This correlates well with the frequency of 10-23% reported in various Indian studies [17,18,27-30], but it is higher than the 6-8% reported in Western countries.

TOF was the most common cyanotic CHD, comprising 16%, correlating well with other studies [19,30]. Out of the 201 children in our study, 82(40.8%) underwent surgery, while the rest were managed conservatively with regular follow-ups. Out of 82, 54(65%) were under one year of age.

Overall mortality, on one-year follow-up, in these children was 36(17.9%). 10(27.7%) children underwent surgery (palliative or definitive) but still could not survive. Most (35) of the deaths (97.2%) occurred in the infant age group. The mean age (in months) of children (at diagnosis) in the expired group was 1.9722 ± 2.90 compared to 16.45 ± 29.29 in the survival group, and this was highly significant (p=0.01) as shown in Table 4.

**Table 4:** Age at diagnosis and survival correlation N: number of children; SD: standard deviation; SEM: standard error of the mean *Significance at 1% level of probability

Outcome		N	Confidence interval	SD	SEM	t-stat	p-value
Age at diagnosis	Survival	165	16.458±4.043	29.29492	2.280	2.958	0.01*
	Expired	36	1.972±0.400	2.90368	0.484		

Strength and limitations

This is the first study done on congenital heart defects with a considerable number (201) over five years in these far-flung islands. However, it is a single-center, hospital-based review and not a community-based study. Therefore, the study does not provide information on the prevalence of congenital heart diseases in the general pediatric population of this place. Furthermore, it is a retrospective review of the medical case records, which may contain some incomplete data or missing information. Diagnostic tools like cardiac catheterization or cardiac computed tomography (CT) scans were not available at Port Blair during the time of the study period and cardiac diagnoses were mainly based on echocardiographic findings.

## Conclusions

The profile of various CHDs in our study was largely similar to pre-existing literature. Although most of the CHDs were detected during infancy, a higher proportion of complex lesions in our study group resulted in adverse outcomes, even in surgically managed cases. The need of the hour is to undertake further studies on the probable etiology of CHD in this population.

Also, recommending routine use of fetal echocardiography may permit antenatal termination of such complex heart defects. Also, suggestions to set up a cardiothoracic center in these islands may help better and more timely palliative and definitive management of complex heart lesions.
